# Born to survive: how cancer cells resist CAR T cell therapy

**DOI:** 10.1186/s13045-021-01209-9

**Published:** 2021-11-22

**Authors:** Jean Lemoine, Marco Ruella, Roch Houot

**Affiliations:** 1 Department of Hematology, AP-HP, Université de Paris, Paris, France; 2 Center for Cellular Immunotherapies and Division of Hematology-Oncology, University of Pennsylvania, Philadelphia, PA USA; 3 Department of Hematology, CHU de Rennes, Université de Rennes, INSERM U1236, 2 rue Henri Le Guilloux, 35033 Rennes Cedex 9, France

**Keywords:** Immunotherapy, Lymphoma, Leukemia, Myeloma, Therapy

## Abstract

Although chimeric antigen receptor T cells demonstrated remarkable efficacy in patients with chemo-resistant hematologic malignancies, a significant portion still resist or relapse. This immune evasion may be due to CAR T cells dysfunction, a hostile tumor microenvironment, or resistant cancer cells. Here, we review the intrinsic resistance mechanisms of cancer cells to CAR T cell therapy and potential strategies to circumvent them.

## Introduction

**Fig. 1:**
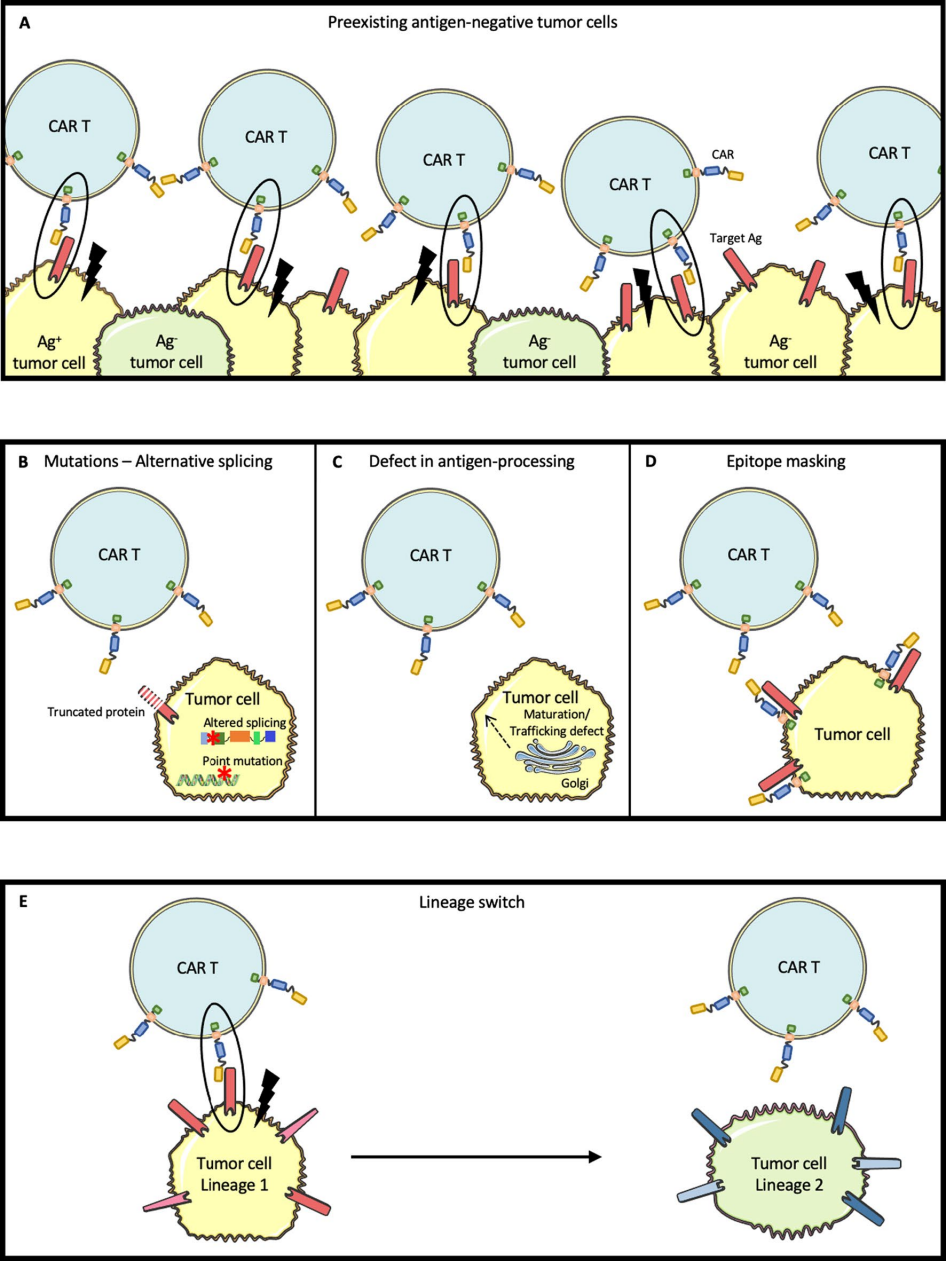
Mechanisms responsible for loss of target antigen, conferring resistance to CAR T cell therapy. **A** Due to tumor heterogeneity before any treatment, pre-existing antigen-negative tumor cells may be responsible for resistance to CAR T cell therapy. **B** Point mutations or altered alternative splicing may lead to a truncated target antigen that can no longer be recognized by CAR T cells. **C** Defect in target antigen maturation and trafficking due to lack of appropriate chaperon proteins may be responsible for target antigen membrane expression loss. **D** Exceptionally, a tumor cell may be transfected with the CAR vector leading to an epitope masking by the CAR itself and hiding the target antigen from the CAR T cells. **E** Lineage switch may be responsible for a complete phenotypic markers remodeling including loss of the target antigen

**Fig. 2:**
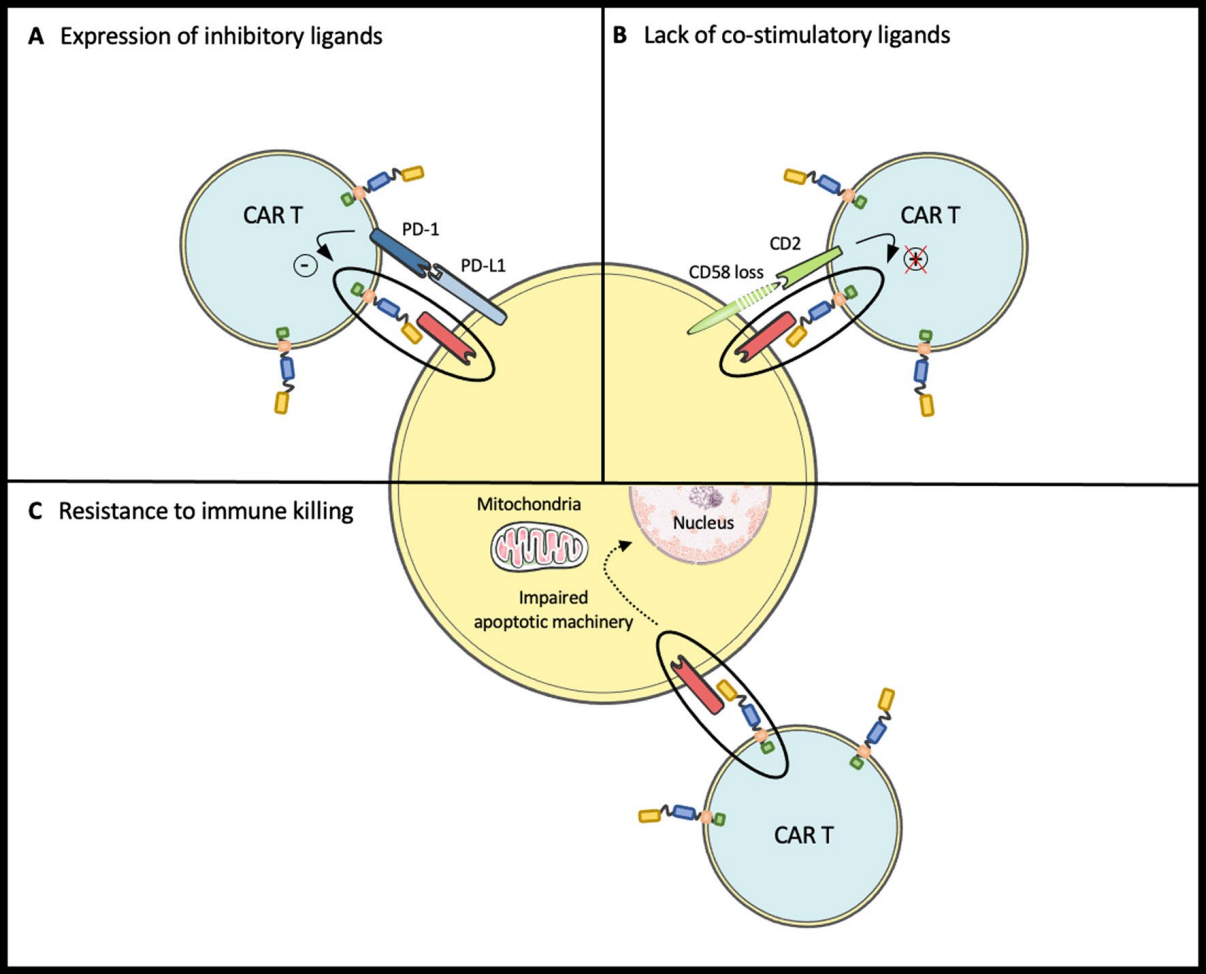
Resistance mechanisms to CAR T cell therapy independent of target antigen loss. **A** Expression of inhibitory ligands (such as PD-L1) by tumor cells inhibit CAR T cell cytotoxicity despite target antigen recognition by CAR. **B** Lack of CD58 expression by tumor cells prevent CD2 to deliver a costimulatory to CAR T cell resulting in an impaired cytotoxicity despite target antigen recognition by CAR. **C** Impaired apoptotic machinery in tumor cells confer intrinsic tumor cell resistance to CAR T cell mediated immune killing despite target antigen recognition by CAR

In the last few years, chimeric antigen receptor (CAR) T cell therapy has emerged as a novel therapy for the treatment of B cells malignancies. Remarkably, CAR T cells can rescue patients who have failed multiple lines of therapies. Thus far, CAR T cells have been approved by the food and drug administration (FDA) for the treatment of B cell acute lymphoblastic leukemia (B-ALL), diffuse large B cell lymphoma (DLBCL), mantle cell lymphoma (MCL), follicular lymphoma (FL) and multiple myeloma (MM). Despite this progress, a significant portion of patients still experience primary or secondary resistance to this treatment [1]. Resistance mechanisms to CAR T cell immunotherapy can involve the CAR T cells, the tumor microenvironment, or the cancer cells (Table 1) [2]. The current review focuses on cancer cells’ intrinsic mechanisms of resistance to CAR T cells therapy, which include loss of the target antigen (Ag), expression of inhibitory receptors, lack of costimulatory ligands, and resistance to immune killing (Figs. 1 and 2). For each of these resistance mechanisms, we also discuss potential strategies which are envisioned to circumvent them (Table 2).

**Table 1:** Mechanisms of resistance to CAR T cell therapy

	Mechanisms of resistance	References
CAR T cells	Lack of expansion Lack of persistence Defective effector function (exhaustion)	[7, 8, 94] [7, 8, 94] [95, 96]
Tumor microenvironment	Impaired trafficking Metabolism/Hypoxia Immune suppression: Immunosuppressive cells (stroma, myeloid cells, regulatory T cells) Immunosuppressive cytokines (TGF-b, IL-10, IL-35)	[97–101] [102–104] [105, 106]
Tumor cells	Loss of target antigen Expression of inhibitory ligands (PD-L1 expression) Lack of costimulatory ligands (CD58 loss) Resistance to immune killing	[3–10, 12–22] [59–63] [75–77] [78–80]

**Table 2:** Therapies envisioned to circumvent resistance mechanisms due to cancer cells

Mechanism	Treatment	
Loss of target-Ag	CAR-T construct	Higher affinity scFvs [35, 36] Hinge region [36]
	Target multiple antigens	CAR T cells with different specificities [37, 39–43] Tandem or bicistronic CARs [44–49]
	Target tumor cells independently of the CAR T target	“Armored” CAR T cells [50–54] TRAIL-mediated death (low-dose radiation) [55]
	Reducing antigen loss on target cell surface	γ-secretase inhibitors [57]
	Deplete transduced tumor cells in case of epitope masking	Anti-CAR19 idiotype CAR T cells [58]
Expression of inhibitory ligands (PD-L1)	Combination with CPI	CAR T cells + anti-PD1 Ab [66–68] Anti-PD1-secreting CAR T cells [69]
	PD-L1-resistant CAR T cells	PD1-KO [70–72] PD1 dominant-negative receptor [73] PD1 switch receptor [74]
Lack of costimulatory ligand (CD58)	Provide CD2 costimulation independently of CD58	CAR with a CD2 signaling domain [77]
Resistance to immune killing	Enhance expression of death receptors	DNA damaging agents [81] Histone deacetylase (HDAC) inhibitors [82, 85] Proteasome inhibitors [83] Cyclooxygenase-2 (COX2) inhibitors [84, 85]
	Enhance sensitivity to CAR T cell killing	Smac mimetics (IAP inhibitors) [80] Bcl2 inhibitors [80, 86, 87]

## Loss of target-Ag

Thus far, loss of target-Ag has been the most widely studied mechanism by which cancer cells may resist or escape CAR T cell therapy (Fig. 1) [3–5]. Relapses with a target-Ag-negative clone may occur by immune-editing resulting in the selection of a pre-existing Ag-negative subclone, or possibly by acquired loss of the target-Ag that was initially expressed by the tumor cells.

### Incidence

The incidence of Ag-loss after CAR T cell therapy may vary across histology. In B-ALL, CD19-loss accounts for approximately 7–25% of relapses after CAR T cells [6–10]. CD19-negative relapses seem to be associated with high tumor burden at the time of lymphodepletion [11]. In DLBCL, approximately a third of relapses exhibit CD19 loss or downregulation [12–18]. Importantly, CD19-negative or -low leukemia or lymphoma cells retain expression of other B cell markers such as CD22 for B-ALL, CD20 and CD79a for lymphoma [18]. In MCL, Wang and colleagues reported 14 (23%) relapses among the 60 patients in the primary efficacy analysis of the ZUMA-2 trial, of which 1 (7%) had undetectable CD19 at relapse [19]. In FL, all 13 patients with evaluable tumor biopsies at progression in the ZUMA-5 trial had detectable CD19 [20]. In MM patients treated with CAR T cells against B cell maturation antigen (BCMA), 1 (8%) out of 12 treated patients exhibited biopsy-proven BCMA-loss at relapse, 8 patients remained BCMA-positive, and 3 were not evaluable for BCMA expression (no biopsy at relapse/progression) [21]. In another cohort, of 18 subjects with evaluable serial BCMA expression after BCMA CAR T cells infusion, 12 (67%) had a decline in BCMA intensity on myeloma cells, including 4 out of 9 non-responders [22].

### Mechanisms

Several mechanisms have been reported to explain Ag-negative relapses, including selection of pre-existing Ag-negative tumor cells, mutation or splicing-variation, altered maturation/trafficking affecting target-Ag expression, epitope masking, and lineage switch/transdifferentiation (Fig. 1). Most data regarding the mechanisms of Ag-loss come from the clinical experience of CD19 CAR T cells in pediatric B-ALL. Flow cytometry analysis of 628 cases of relapsed or refractory (R/R) B-ALL from the Children’s Hospital of Philadelphia (CHOP) revealed that, before any treatment, about 17% of cases had more than 1% of CD19-negative tumor cells. Moreover, 7% of patients had a decreased expression of CD19, and about a quarter had low-normal CD19 expression [23]. Thus, a significant number of patients have pre-existing tumor cells with absent or low membrane expression of target-Ag which can be selected upon immune pressure from CAR T cells. Such CAR T cell-mediated immunoediting shapes the initial tumor heterogeneity favoring the emergence of target-Ag-negative relapses [24]. Furthermore, prior exposure to the CD19-directed, bispecific T cell engager (BiTE), blinatumomab was shown to be associated with a significantly higher rate of CD19-negative relapses after CAR T cell therapy [25].

#### Mutations

Flow cytometry analysis of 17 samples from pediatric and young-adult patients with R/R B-ALL showed that 12 patients had CD19-negative disease [7]. In all 12 patients, mutations in the CD19 domain were identified. These mutations were responsible for a truncated protein with a nonfunctional or absent transmembrane domain [26]. Such mutations affecting CD19 were also reported in the context of refractory DLBCL treated with CD19 CAR T cells [27] (Fig. 1B).

#### Alternative splicing

Sotillo and colleagues identified alternative splicing as a mechanism of Ag-loss by comparing CD19-negative samples at relapse with CD19-positive samples before CAR-T treatment from the same patients [28]. They found several CD19 splice variants expressed by B-ALL, especially affecting exon-2, resulting in the loss of the extracellular epitope of CD19 which is recognized by the CAR T cells. In other cases, alternative splicing resulted in lack of transmembrane domain precluding CD19 expression on the cell surface. Recent data reported the existence of CD19 isoforms at diagnosis which lack the CD19 epitope recognized by CAR T cells. This subclone may evolve as a dominant clone during CAR therapy and promote Ag-negative relapses [29] (Fig. 1B).

#### Defect in Ag-processing

Defect in maturation and trafficking of CD19 has been reported as a cause of resistance to CD19/CD3 BiTE, blinatumomab [30]. CD81 is a chaperone protein that regulates CD19 protein maturation and trafficking from the Golgi to the cell surface. In a patient, post-transcriptional regulation was responsible for CD81 loss, thus precluding CD19 processing and maturation in the Golgi. This alteration resulted in Ag-negative relapse 19 months after completion of blinatumomab treatment for B-ALL. This mechanism of resistance, reported with BiTE, may also be shared with CAR T cells, although not yet described (Fig. 1C).

#### Epitope masking

Ruella and colleagues reported the insertion of the CAR transgene into a single leukemic B cell during CAR T cell manufacturing. Expression of the CAR at the blast surface masked the CD19 epitope and thereby prevented the recognition of the tumor cell by CAR T cells. This rare event has been observed in a single patient with B-ALL who relapsed 9 months after treatment with CD19 CAR T cell [31]. Most CAR T manufacturing protocols now include T cell selection to avoid this potential complication (Fig. 1D).

#### Lineage switch and transdifferentiation

Ag-loss may also be due to a lineage switch [32]. Unlike other mechanisms previously described, lineage switch is not only responsible for CD19 loss but results in a broader phenotypic switch resulting in the acquisition of acute myeloid leukemia (AML) markers. Two patients with MLL-rearranged B-ALL treated with CD19 CAR T cells on the SCRI trial [33] and one patient from the Fred Hutchinson Cancer Center [10] were reported to experience a lineage switch with CD19-negative escape after CD19 CAR T cell therapy. As an exceptional event, MCL transdifferentiation into poorly differentiated sarcoma has been described after CD19 CAR T cells in one patient [34]. This transdifferentiation process was associated with a profound reprogramming of the epigenome responsible for tumor progression two months after CAR T cell infusion (Fig. 1E).

### Strategies to overcome Ag-low/negative relapses

Ag-low relapses may be prevented by increasing the sensitivity of CAR T cells to their target whereas Ag-negative relapses may be overcome by targeting multiple Ags or by killing tumor cells in an Ag-independent manner. In both cases, strategies meant to re-induce or increase Ag-expression may also be beneficial (Table 2).

#### CAR T cell constructs with enhanced sensitivity to prevent low-Ag relapses

CAR T cells with higher affinity single-chain variable fragments (scFvs) have been developed to recognize Ags expressed at a low density. In vitro, CAR T cells harboring a high-affinity scFv were able to recognize their target when expressed at very low levels, including when the target was undetectable by flow cytometry [35]. Furthermore, adding immunoreceptor tyrosine-based activation motifs (ITAM) enhanced the strength of intracellular signaling, both in CD28 and 4-1BB CAR T cells, and thus enabled recognition of tumor cells with a low Ag-density [36]. Finally, the hinge region may impact the ability of CAR T cells to bind its epitope. As an example, replacement of the CD8 hinge-transmembrane region of a 4-1BBζ CAR with a CD28-hinge-transmembrane region lowered the threshold for CAR reactivity and enhanced the killing of CD19-low leukemic cells [36]. These approaches may be particularly beneficial in the context of CD22 and BCMA CAR T cells because patients often relapse with CD22dim [37, 38] or BCMAdim [22] upon these immunotherapies whereas relapses upon CD19 CAR T cells mostly exhibit complete CD19 loss.

#### CAR T cells targeting multiple Ags to prevent Ag-negative relapses

As discussed above, recent data suggest that prior to any treatment, a significant portion of patients harbor pre-existing Ag-negative tumor cells. This initial tumor heterogeneity may lead to relapse upon CAR T cell therapy. To overcome Ag-negative relapses, CAR T cells directed against multiple Ags have been developed [39–41]. This may be achieved by infusing a cocktail of CAR T cells with unique but different specificities or by infusing CAR T cells with multiple specificities.

CAR T cells with different specificities can be administered simultaneously or sequentially to prevent Ag-negative relapses. In a phase I trial, 21 B-ALL patients were treated with CD22 CAR T cells including 17 who had been previously treated with a CD19-directed immunotherapies [37]. CD22 is expressed in most cases of B-ALL and is usually retained following CD19 loss. In this study, all 5 patients with a CD19dim or CD19-negative B-ALL achieved a complete remission after receiving > 1 × 10^6^ CD22 CAR T cells. The median duration of response was 6 months. Interestingly, relapses were associated with decreased CD22 expression by tumor cells. Similarly, Baird et al. reported 3 patients with CD19 CAR resistant DLBCL who were treated with CD22 CAR T cells and achieved a complete remission [42]. From these observations, Pan and colleagues hypothesized that a sequential administration upfront of 2 CAR T cell products targeting different Ags may improve long-term outcomes. They conducted a phase 1 trial evaluating a sequential administration of CD19 and CD22 CAR T cells in pediatric patients with R/R B-ALL [43]. In this trial, 17 (85%) of the 20 patients treated with sequential CAR T cells remained in CR at the end of the study and 3 relapsed at 6.6, 6.9 and 11.4 months, resulting in a 1-year PFS and OS of 79.5% and 92.3%, respectively. Among relapsed patients, one exhibited CD22 downregulation on leukemic cells and CD19 Ag-loss was observed in 2 patients.

CAR T cells with multiple specificities have also been developed which can be i) tandem (or bivalent) CARs with two distinct Ag-binding sites on a single extracellular domain, or ii) bicistronic CARs which are engineered using a single vector encoding two distinct CARs to allow dual targeting through separate extracellular motifs. CD19/CD22 tandem CAR constructs were administered to 12 patients with R/R B-ALL: 11 achieved a CR and 1 experienced primary progressive disease with CD19 retention [44]. In a recent study focusing on adult R/R B-ALL, 6 out of 6 infused patients achieved an MRD-negative CR after infusion with tandem CD19/CD22 CAR T cells. Of note, one patient experienced a relapse at 5 months post-infusion with CD19-negative and CD22-low blasts [45]. Such strategies have also been applied to R/R aggressive B lymphomas. In a phase I dose-escalation study, 11 patients (5 DLBCL, 4 MCL, 2 CLL) were treated with CD19/CD20 tandem CAR T cells [46]. The ORR was 82% at day 28 (6/11 CR and 3/11 PR). All progressing patients retained either CD19 or CD20 positivity suggesting alternative resistance mechanisms to Ag-loss. Toxicity was acceptable [47]. The AUTO3 trial evaluated a bicistronic CAR T cell targeting both CD19 and CD22 in pediatric R/R B-ALL. All 7 evaluable patients achieved a CR/CRi following CAR T cells infusion with a negative minimal residual disease (MRD) [48]. Three relapses were reported including one with CD19 negative/CD22 low expression. Thus, Ag escape may be observed as a resistance mechanism even upon multi-targeted strategies. This bicistronic construct has also been evaluated in the phase I Alexander study for the treatment of R/R DLBCL, combined with the anti-PD1 antibody, pembrolizumab [49]. In this study, among the 11 patients treated at a dose > 50 × 10^6^, the ORR and CRR were 64% and 55%, respectively. Toxicities were acceptable and in line with CAR T cells targeting a single Ag.

#### CAR T cells to kill cancer cells in an Ag-independent manner

“Armored” CAR T cells have been developed to secrete immune stimulatory cytokines (e.g., IL12 or IL18) or express costimulatory ligands (e.g., CD40L, Flt3L, or 4-1BBL) in order to enhance CAR T cell efficacy (“auto/transactivation”) and/or generate/recruit natural antitumor T cells (“epitope spreading”).

Armored CAR T cells secreting IL12 and IL18 demonstrated enhanced activity in preclinical models. IL12 secretion by CAR T cells resulted in increased antitumor efficacy [50]. IL12 can increase the antitumor function of CAR T cells in an autocrine manner. It can also shape the tumor microenvironment, rendering CAR T cells resistant to regulatory T cells and myeloid-derived suppressor cells immunosuppression. Avanzi and colleagues designed armored IL18-secreting CAR T cells, which increased IFN-γ secretion and tuned the tumor microenvironment toward an IFN-γ signature [51]. These IL18-secreting CAR T cells were able to modulate the tumor microenvironment and enhance the endogenous antitumor immune response.

Kuhn and colleagues developed CAR T cells expressing CD40L constitutively. This construct enables CD40L expressed on the CAR T cells to engage with CD40-positive tumor cells (resulting in direct cytotoxicity) and with antigen-presenting cells (APC). CD40L-CD40 interaction triggered activation of tumor-adjacent APC and increased expression of costimulatory molecules such as CD40, CD86 and major histocompatibility complex (MHC) class II. Thus, constitutive expression of CD40L by CAR T cells can generate endogenous antitumor T cells which can recognize and kill tumor cells through their TCR, thereby preventing the risk of immune escape via the loss of a single Ag [52]. Similarly, Lai et al. engineered T cells to secrete the dendritic cell (DC) growth factor Fms-like tyrosine kinase 3 ligand (Flt3L) [53]. This construct induced the activation of endogenous T cells, enabling a broader repertoire of tumor Ags to be targeted via the expansion of intratumoral APCs, significantly improving tumor responses.

Costimulatory ligands such as CD80 and 4-1BBL may also be expressed on CAR T cells to stimulate bystander endogenous and CAR T cells (transactivation). CD80 and 4-1BBL bind to two costimulatory receptors expressed on T cells, CD28 and 4-1BB, respectively. Primary human T cells overexpressing CD80 and 4-1BBL were shown to eradicate tumor cells very efficiently, even in the absence of costimulatory ligands [54]. CD80/4-1BBL expressing CTL were able to induce trans-costimulation of bystander T cells. These strategies could be applied to CAR T cells. By stimulating endogenous T cells locally, they may promote/enhance tumor cell killing beyond their target-Ag.

Alternative death signaling pathways may also be used to enable CAR T cells to kill cancer cells independently of their target. For instance, low-dose radiation induces expression of death receptors such as TRAIL-receptors on the tumor cells’ surface. Thus, tumor cells are sensitized to TRAIL-mediated apoptosis by CAR T cells. CAR T cells activated by Ag-positive tumor cells can then induce bystander killing of Ag-negative tumor cells through the death receptors pathway. This strategy increased both on-target and bystander CAR T cell cytotoxicity, enabling tumor control even in the case of Ag-loss variants [55].

#### Reducing Ag-loss on target cell surface

Although CAR T cells may exert their action independently of their target-Ag, their efficacy is thought to be tightly associated with the density of target-Ags on the cell membrane. Thus, combining CAR T cells with treatments that increase target expression on the tumor cells could be of interest. Efficacy of BCMA CAR T cells for the treatment of multiple myeloma is thought to be limited by BCMA cleavage from the tumor cell surface by a γ-secretase, which decreases ligand density on myeloma cells [56]. In murine models, inhibition of the γ-secretase activity reduced Ag-loss on the target cell and improved antitumor efficacy of BCMA CAR T cells [57]. Based on these data, clinical trials evaluating the combination of γ-secretase inhibition with concurrent BCMA CAR T cell treatment are ongoing (NCT03502577).

#### Depleting tumor cells in case of epitope masking

Unintentional transduction of B-ALL blasts during CD19 CAR T cells manufacturing can lead to CD19 CAR T cells treatment resistance through epitope masking. Anti-CD19 CAR idiotype CAR has been developed to specifically recognize and deplete transduced B-ALL blasts [58].

## Expression of inhibitory ligands (PD-L1 expression)

The programmed death-1 (PD-1)/programmed death-1 ligand-1 (PD-L1) axis is a well-known immune checkpoint inhibitor pathway. The inhibitory ligands PD-L1 and PD-L2 may be expressed by tumor cells or their microenvironment. These ligands prevent T cell activation upon binding to their receptor (i.e., PD-1), allowing immune escape (Fig. 2A).

### Incidence

PD-L1/L2 have been reported to be expressed in B cell malignancies. In R/R DLBCL, 16% of all evaluable samples exhibited low-level copy gain and 3% had amplification of 9p24.1, the locus encoding for PD-L1 and PD-L2 [59, 60]. By immunohistochemistry, 4 out of 46 cases (9%) showed membrane expression of PD-L1 on biopsy specimens. Primary mediastinal large B cell lymphoma (PMBCL) has been reported to be frequently associated with genetic aberrations at 9p24, resulting in tumor expression of PD-L1 and PD-L2 [61]. Finally, PD-L1 has also been reported to be expressed in a subset of B-ALL [62, 63].

### Mechanisms

PD-1 is expressed on activated CAR T cells [64]. PD-L1 expression by the tumor cells or the tumor microenvironment may inhibit CAR T cell cytotoxicity and induce immune resistance. Neelapu et al. demonstrated that 13 (62%) out of 21 DLBCL patients who had progressed after Axi-Cel therapy in the ZUMA-1 trial expressed PD-L1[65]. Hence, unleashing the inhibition of immune checkpoints such as PD-1 may enhance the efficacy of CAR T cell therapy.

### Strategies to overcome PD-L1-mediated resistance

Several strategies may be envisioned to overcome PD-L1-mediated immune suppression, including combination of CAR T cells with anti-PD1/PD-L1 antibodies, anti-PD1-secreting CAR T cells, or PD-L1-resistant CAR (Table 2).

#### Combination of anti-PD1/PD-L1 antibodies with CAR T cells

The combination of an immune checkpoint blocker with CAR T cells has been evaluated in 2 different setting: i) at the time of relapse after CAR T cell therapy and ii) at the time of CAR T cell infusion (i.e., upfront). Thus far, only small cohorts of patients have been treated with this combination. In a single-center study, 12 DLBCL patients in relapse or progression after CD19 CAR T cells received an anti-PD1 antibody (Ab), pembrolizumab [66]. Among 11 evaluable patients, 1 achieved a complete response, 2 a partial response, 1 a stable disease, and 7 progressed. Interestingly, 9 of the 12 patients showed a re-expansion of the CAR T cells as measured by transgene copy number. However, no correlation could be made between the peak of CAR T cells and the clinical response in this small cohort. Anti-PD1 antibodies have also been combined with CAR T cells at the time of adoptive transfer. In a small study, 6 DLBCL patients received the anti-PD-L1 Ab durvalumab 21–28 days after CAR T cells infusion, and 9 patients were treated with durvalumab the day before infusion [67]. The overall response rate was 50%. With a median follow-up of 10.6 months, only one patient among the 5 patients who achieved a CR has relapsed. In this study, 5/13 patients developed a cytokine release syndrome (CRS) including one grade 4, and one patient experienced a neurotoxicity. In the ZUMA-6 study, CD19 CAR T cells were given in combination with the anti-PD-L1 Ab atezolizumab for the treatment of R/R DLBCL [68]. In this study, atezolizumab was administered starting either on day 21, 14 or 1 post CAR T cell infusion. The most common grade 3–4 AEs were anemia (9/12), encephalopathy (5/12), and neutropenia (5/12). Grade 3–4 CRS and neurotoxicity occurred in 3/12 and 6/12 patients, respectively. After a median follow-up of 4.4 months, 9 out of 10 patients experienced an objective response, including 6 CR and 3 PR. The ALEXANDER trial evaluated the combination of a bispecific CD19/CD22 CAR T cell (Auto3) and pembrolizumab [49]. Among 11 patients treated at a dose > 50 × 10^6, the ORR and CRR were 64% and 55%, respectively. At this dose level, there were no case of severe CRS nor neurotoxicity of any grade. These observations indicate that combination strategies of immune checkpoint blockers with CAR T cells may be beneficial for a subset of patients and suggest that other resistance pathways might play a crucial role.

#### Anti-PD1-secreting CAR T cells

Rafiq and colleagues developed “armored” CAR T cells capable of secreting PD1 blocking scFv which can act both in a paracrine and autocrine manner [69]. In murine models, the efficacy of these CAR T cells against PD-L1-expressing tumors was at least as good as the combination of CAR T cells with an anti-PD1 Ab. Such CAR T cells allow local delivery of anti-PD1 scFv which could limit the toxicities of a systemic exposure to checkpoint inhibitors.

#### PD-L1-resistant CAR T cells

CAR T cells have been engineered to be resistant to PD-1 signaling. This can be achieved by knocking-out the PD1 gene using the CRISR-Cas9 technology [70–72] or by transducing a PD1 dominant-negative receptor [73]. CAR T cells can also be transduced with a PD1/CD28 chimeric switch receptor. This receptor contains the extracellular domain of PD1 fused with the transmembrane and cytoplasmic domain of the costimulatory molecule, CD28. Ligation of PD-L1 to its receptor (PD1) transmits an activating signal (via the CD28 cytoplasmic domain) instead of the inhibitory signal normally transduced by the PD1 cytoplasmic domain [74]. These CAR T- cells have been shown to resist PD1 mediated inhibition in preclinical models. However, no clinical data are yet available with these constructs.

## Lack of costimulatory ligand (CD58 loss)

CD58 is the ligand for the CD2 molecule expressed on human T cells. CD2 provides a costimulatory signal for T cell proliferation, cytokine production and activation via TCR signaling [75, 76]. Similar to natural cytotoxic T cells, CD2 ligation is crucial for CAR activation and cytoskeletal rearrangement required for tumor cell killing (Fig. 2B).

### Incidence

CD58 alteration (mutation or loss of expression) is seen in approximately a quarter of DLBCL. Furthermore, CD58 alteration is associated with a worse outcome after CD19 CAR T cell treatment [77].

### Mechanisms

CD58 alteration may be due to mutations or lack of expression as measured by IHC [77]. CD58 alteration decreases CAR T cell activation and cytotoxicity in preclinical models.

### Strategies to overcome lack of costimulatory ligands

Lack of CD58-CD2 signaling may be overcome by adding a second CAR construct containing a CD2-signaling domain integrated in the cytoplasmic tail of the CAR (Table 2). CAR binding to its target-Ag will induce CD2 signaling independently of CD58 expression by the tumor cells. Such construct demonstrated increased activity in preclinical models but have not yet been tested in patients [77].

## Resistance to immune killing

Tumor cells may be resistant to immune cell killing by CAR T cells by a mechanism called “intrinsic resistance” (Fig. 2C). This specific mechanism of resistance has been recently reviewed in details elsewhere [78].

### Incidence

The intrinsic resistance of tumor cells to CAR T cell killing has been recently reported. However, its prevalence in hematologic malignancies remains unknown.

### Mechanisms

Recently, accumulating data revealed that impaired apoptosis machinery in tumor cells could render tumor cells resistant to immune killing by CAR T cells. Two unbiased genome-wide loss-of-function screens in B-ALL and B cell lymphoma cell lines revealed the crucial role of the death receptor pathway of apoptosis to mediate CAR T cell cytotoxicity [79, 80]. These studies demonstrated that disruption of genes associated with pro-apoptotic death receptor signaling pathway such as FADD, BID, CASP8, and TNFRSF10B conferred resistance to CAR T cell killing. Conversely, knock-out of anti-apoptotic genes such as CFLAR, TRAF2, and BIRC2 led to an increased susceptibility to CAR T cell killing. Using samples from two multicenter trials of relapsed/refractory pediatric and adult B-ALL, Singh and colleagues found a significantly higher death receptor signaling signature in samples from patients who had achieved complete remissions compared to patients who did not respond despite retaining CD19 expression [79].

### Strategies to overcome intrinsic resistance to CAR T cell killing

Based on these observations, strategies have been considered to sensitize tumor cells to death receptor mediated apoptosis (Table 2). Lowering the threshold necessary to induce this type of cellular death may indeed overcome intrinsic resistance of tumor cells to CAR T cell therapy. Several cancer therapeutic agents may modulate death receptor expression by tumor cells including DNA damaging agents [81], histone deacetylase (HDAC) inhibitors [82], proteasome inhibitors [83] and cyclooxygenase-2 (COX2) inhibitors [84]. In preclinical models, HDAC inhibitors (SAHA and LBH589) and COX2 inhibitors (celecoxib) were able to partially reverse CD19 CAR T cell resistance of non-Hodgkin lymphoma cell lines due to altered death receptor pathway machinery [85]. To identify novel candidate molecules, Dufva and colleagues carried out a high-throughput drug screen using a coculture assay with CD19-directed CAR T cells in presence of the CD19-positive B-ALL cell line, NALM6. Strikingly, the three drugs that most significantly enhanced CAR T cell cytotoxicity all belonged to the same pharmacological class, namely second mitochondrial-derived activator of caspases (Smac) mimetics or inhibitor of apoptosis proteins (IAP) inhibitors (Birinapant, AT-406, and LCL-161) [80]. Another pharmacologic class which may be of interest are the B cell lymphoma 2 (Bcl-2) inhibitors. Bcl-2 prevents apoptosis. Its overexpression can promote tumor cells survival and may lead to treatment resistance. Thus, the combination of Bcl-2 inhibitors with CAR T cells may be an attractive strategy. In a coculture model containing B-ALL and CD19 CAR T cells, Bcl-2 inhibition decreased the apoptosis threshold in leukemic cells leading to an enhanced CAR T cell cytotoxicity [86]. Moreover, in vitro pre-sensitization of B-ALL cells with the Bcl-2 inhibitor venetoclax resulted in an enhanced CAR T cell mediated cytotoxicity by upregulating the CD19 expression and pro-apoptotic proteins [87]. However, these strategies are yet to be tested in clinic.

## Conclusion

Understanding how tumor cells resist CAR T cell therapy is a crucial step toward the development of strategies to improve CAR T cell efficacy. Fundamental and clinical studies on evasions to monoclonal antibody treatment were instructive as some resistance mechanisms are shared with CAR T cells given that both treatments are responsible for a selection pressure on a specific tumor marker. Indeed, loss of target antigen was previously reported as a resistance pathway to monoclonal antibodies or BiTE as rituximab for CD20, blinatumomab for CD19 and inotuzumab ozogamicin for CD22 [30, 88–90]. Thus, in the CAR T cell field, loss of target-Ag has been the best-characterized mechanism of resistance, and new approaches are being developed to overcome or prevent this issue, including CAR T cells directed against multiple Ags. Recent studies unveiled other resistance mechanisms developed by cancer cells to evade eradication by CAR T- cells, including expression of inhibitory ligands, lack of costimulatory ligands, and intrinsic resistance to CAR-T killing. Each of these resistance mechanisms may be circumvented by strategies directed toward the CAR T cells or the tumors cells. However, most of these strategies remain to be evaluated in patients. From a broader perspective, one shall remember that three compartments may contribute to CAR T cell resistance: the tumor cells (reviewed here), the tumor microenvironment, and the CAR T cells. The role of tumor microenvironment may be particularly important in solid tumors due to the extracellular matrix and cytokine milieu present in non-hematopoietic tissues [91–93]. Thus, novel CAR T cell designs should address the resistance mechanisms of all three compartments. Finally, as this field is moving forward, it will become increasingly important to characterize the resistance mechanisms of each individual tumor in order to personalize CAR T cell therapy with the optimal product or combination.

## Data Availability

Not applicable.

## Abbreviations

CAR: Chimeric antigen receptor
FDA: Food and drug administration
B-ALL: B cell acute lymphoblastic leukemia
DLBCL: Diffuse large B cell lymphoma
MCL: Mantle cell lymphoma
FL: Follicular lymphoma
MM: Multiple myeloma
Ag: Antigen
BCMA: B cell maturation antigen
R/R: Relapsed or refractory
CHOP: Children’s Hospital of Philadelphia
BiTE: Bispecific T cell engager
AML: Acute myeloid leukemia
scFvs: Single-chain variable fragments
ITAM: Immunoreceptor tyrosine-based activation motifs
MRD: Minimal residual disease
APC: Antigen-presenting cells
MHC: Major histocompatibility complex
DC: Dendritic cell
Flt3L: Fms-like tyrosine kinase 3 ligand
PD-1: Programmed death-1
PD-L1: Programmed death-1 ligand-1
PMBCL: Primary mediastinal large B cell lymphoma
Ab: Antibody
CRS: Cytokine release syndrome
HDAC: Histone deacetylase
COX2: Cyclooxygenase-2
Smac: Second mitochondrial-derived activator of caspases
IAP: Inhibitor of apoptosis proteins
Bcl-2: B cell lymphoma 2
